# Modelling the temporal dynamics of *Microsporidia MB* prevalence in *Anopheles* mosquitoes under environmental variability

**DOI:** 10.1038/s41598-026-47368-y

**Published:** 2026-04-10

**Authors:** Charlène N. T. Mfangnia, Matteo Pedercini, Henri E. Z. Tonnang, Jeremy K. Herren

**Affiliations:** 1 Department of Mathematics and Computer Science, Faculty of Science, University of Dschang, P.O. Box : 67, Dschang, Cameroon; 2 International Centre of Insect Physiology and Ecology (icipe), P.O. Box : 30772, Nairobi, 00100 Kenya; 3 Millennium Institute, Washington, DC USA; 4 School of Agricultural, Earth, and Environmental Sciences, University of KwaZulu-Natal, Pietermaritzburg, 3209 South Africa

**Keywords:** *Microsporidia MB*, *Plasmodium* transmission-blocking, Vertical-horizontal transmission, Computer-based simulations, Seasonality, Malaria biocontrol, System dynamics, Diseases, Ecology, Ecology, Zoology

## Abstract

Releasing *Microsporidia MB*-positive mosquitoes is being investigated as a promising component of Integrated Vector Management (IVM) strategies for malaria control. In this study, we developed a System Dynamics model to replicate the observed interactions between humans, mosquitoes, the *Plasmodium* parasite, and the symbiont *MB*. The model accounts for the impact of environmental variables - such as humidity, rainfall, and temperature - on mosquitoes, *Plasmodium*, and *MB*, using exogenous inputs derived from daily climatic observations (rainfall, relative humidity, and temperature) recorded during the mosquito field surveys. We calibrate the model using data collected from Ahero in Kenya, available for 3 years in the past. Model outputs were compared with historical data for two Key Performance Indicators, *KPIs*: adult mosquito abundance and *MB* prevalence in adult mosquitoes. Statistics of fit indicate that the model has good ability to represent the trend for the selected *KPIs*, but not the short-term variation, which is consistent with the objectives of the modelling. Our sensitivity analysis revealed that the equilibrium prevalence of *MB*-positive mosquitoes is more sensitive to the vertical transmission rate, followed by the male-to-female horizontal transmission rate, while the female-to-male transmission rate has less impact. Additionally, we demonstrated that the distribution of the *MB*-intensity within the *MB*-positive mosquito population can drive the extinction or persistence of *MB*-positive mosquitoes. Finally, we evaluated how variations in key climatic drivers (temperature, humidity, and rainfall) influence the prevalence and spread of *Microsporidia MB* in adult mosquito populations using sensitivity analyses of climatic datasets. This study is meant for the design of a decision support tool for adoption by policymakers and implementation of an *MB*-based malaria control intervention.

## Introduction

*Plasmodium* is a parasite that poses a threat to humans as it drives malaria infection, responsible for 610,000 deaths globally in 2024^1^. The parasite is transmitted to humans through the bite of a carrying vector, female *Anopheles* mosquitoes^2^. Malaria control can be either curative, by using drugs when the human is already infected; or preventive, by reducing the contact between the human and the mosquito, or by controlling the vector population. For instance, the use of Insecticide Treated Nets (ITNs), acts both by reducing the contact between the human and the mosquito and by killing the mosquitoes^3^. ITNs interventions have been widely implemented and have contributed to 67–73% of the total 663 million averted malaria cases from 2000 to 2015^4^. Common interventions to control vector population also include Indoor Residual Spray (IRS), which contributes to eliminating mosquitoes at the adult stage; and Larval Source Management (LSM), which contributes to killing mosquitoes at immature stages^4^. ITNs, IRS and LSM are insecticide-based interventions, and thus, their effectiveness is hindered by the emergence of insecticide resistance^5^. As a response to the associated increase in the number of malaria deaths^1^, there is a need for new effective strategies for malaria control.

Vector population replacement using symbionts that block pathogen transmission has emerged as a promising strategy for controlling vector-borne diseases. A prominent example is the release of *Wolbachia*-infected mosquitoes, which has been successfully implemented in several field trials. In Australia, releases resulted in near fixation of the *wMel* strain in *Aedes aegypti* populations^6^, while field deployments in Indonesia and Malaysia have been associated with significant reductions in dengue incidence^7,8^. These outcomes are largely explained by two biological mechanisms: *Wolbachia* infection inhibits viral (dengue virus) development in mosquitoes (*Aedes Aegypti*^9–12^), thereby reducing their transmission capacity, and its cytoplasmic incompatibility phenotype provides a reproductive advantage to infected mosquitoes, facilitating the spread of the symbiont in wild populations following releases^6,9–12^. The potential application of *Wolbachia* for malaria control has also been investigated. Reports have documented the presence of *Wolbachia* in *Anopheline* mosquitoes^13,14^, with some evidence suggesting a protective effect against *Plasmodium* infection^15,16^. However, other studies indicate that *Wolbachia* strains in *An. gambiae s.l.* occur at low densities and exhibit limited vertical transmission efficiency^17^, and some reported detections may result from contamination artifacts^18^. Moreover, naturally occurring *Wolbachia* infections have not been associated with reduced *P. falciparum* development in *An. moucheti* mosquitoes^19^. Clearly, these mixed findings suggest that further research is required to determine whether *Wolbachia* infections in *Anopheles* mosquitoes could be useful for malaria control.

Recently, the *Microsporidia MB* endosymbiont has been shown to block *Plasmodium* development in the mosquito vector^20–27^. *Microsporidia MB* has been detected in *Anopheles* mosquito populations across several African countries, including Kenya^24^, Ghana^21^, Benin^28^, Nigeria^29^, and Niger^29,30^, and has the advantage of being naturally present in populations of *Anopheles* *arabiensis*, *funestus*, *gambiae* and *coluzzi*^20,21,23,24^. In this study, *MB*-positive mosquitoes refer to individuals in which *Microsporidia MB* infection is detected (e.g., through molecular diagnostics), rather than necessarily exhibiting a complete *Plasmodium*-blocking phenotype. According to lab and field experiments in Kenya, the prevalence was initially assessed at an average of 15%^24^; however, studies have shown that prevalence in the field can be highly variable^29^. In addition, *MB* is vertically^24^ transmitted from infected mothers to their progeny and horizontally^20^ transmitted during mating between infected and uninfected individuals. This study aims to investigate the effectiveness of malaria control through the spread of *Microsporidia MB*-positive mosquitoes using system dynamics modelling.

Quantitative modelling of human-vector interaction provides important insights into such complex systems and enables the exploration of intervention policies to support public health planning. System dynamics models have been formulated to address a wide range of health challenges^31^, including vector-borne diseases^32–35^. Focusing specifically on malaria control strategies, the study carried out by^36^ evaluates malaria dynamics while another study by^37^ assessed the costs and benefits of using the DDT insecticide.

Mathematical modelling approaches have been used to investigate the interactive dynamics between *MB*-negative and *MB*-positive mosquitoes^25,38,39^, and effects on malaria incidence. These analyses suggested that the observed low prevalence of *MB*-positive mosquitoes in natural populations may be associated with low male-to-female horizontal transmission efficiency. In addition, several parameters were identified as key determinants of *MB* prevalence, including the efficiencies of vertical and horizontal transmission, the mortality rate of *MB*-positive females, the average mating time after emergence of infected mosquitoes, and the relative attractiveness of *MB*-positive mosquitoes compared with *MB*-negative mosquitoes. Furthermore, the *MB* prevalence to achieve malaria elimination under different local transmission settings in Kenya was also estimated.

However, these models relied on simplified frameworks that did not incorporate several important drivers of real-world transmission dynamics, such as climatic forcing, the different stages of *Plasmodium* development within the mosquito, variability in *MB* density and its associated effects on *Plasmodium* transmission blocking, or mosquito age structure which, together with the extrinsic incubation period (EIP), strongly determines the infectivity period of the mosquito. In addition, potential interactions with other malaria control interventions, such as insecticide-treated nets (ITNs) and indoor residual spraying (IRS), were not considered.

In this work, to address some of the limitations of the previous studies, we employ the system dynamics methodology to build a highly aggregated model that incorporates both human and mosquito dynamics as well as the effects of abiotic factors. Specifically, we structure the mosquito population with respect to life stage, age, *MB*-intensity variability, *Plasmodium* infection stage, and mating stage. The objectives of this work are (1) to understand and reproduce the *MB* dynamics observed in field data, and (2) to provide a practical decision-support tool to test and assess alternative release strategies aimed at reducing malaria incidence.

## Results

### Model performance

**Fig. 1:**
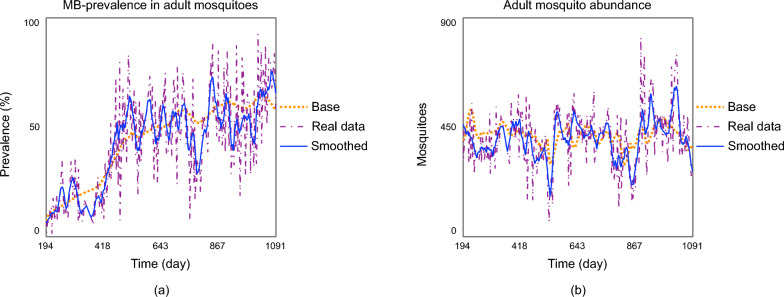
(**a**) *MB*-prevalence in adult mosquitoes and (**b**) adult mosquito abundance. For both graphs, the base is the output of the model run, the smoothed is the 21-day average of the model run and the real data is the historical data.

The assessment of our model’s performance is based on the accuracy of the calibration of the model outcomes to the observed data. The calibration was done both for the adult mosquito abundance and the *MB*-prevalence and the results are presented in Figure 1. The first baseline for the prevalence of *MB*-positive mosquitoes, observed from data collected between July 13, 2021, and May 28, 2024, was used for this calibration.

During the calibration process, we observed that while the model captures the overall temporal trends, it does not reproduce short-term fluctuations in the data. These fluctuations may arise from stochastic events not represented in the model or from measurement errors. To facilitate comparison between model outputs and observed data and better highlight the underlying patterns of behaviour, we introduce a 21-day moving average of the data records, referred to as the “smoothed adult mosquito abundance”. Similarly, the variable “smoothed *MB* prevalence in adult mosquitoes” is defined as a 21-day moving average to better represent the trend in *MB* prevalence. Daily measurements can exhibit substantial variability due to sampling noise; therefore, the 21-day window was selected as a reasonable compromise to reduce short-term fluctuations while preserving the broader temporal dynamics of mosquito populations.

Simulation results in Figure 1 provide a visual confirmation of the model’s ability to reproduce the trends observed in the data. Quantitative analysis of the error further supports this interpretation: the measured *RMSPE* is slightly above 1, reflecting deviations driven primarily by short-term variability, while the bias component (*TBIAS*), calculated based on *Theil’s* statistics, is low (0.6%). This indicates that the model does not exhibit systematic over- or underestimation and that discrepancies are largely associated with fluctuations rather than persistent bias. Overall, these results suggest that the model adequately captures the underlying temporal trends and provides a reliable baseline for evaluating alternative intervention strategies.

### Effect of the distribution of the *MB*-positive mosquitoes according to *MB*-intensity

**Fig. 2:**
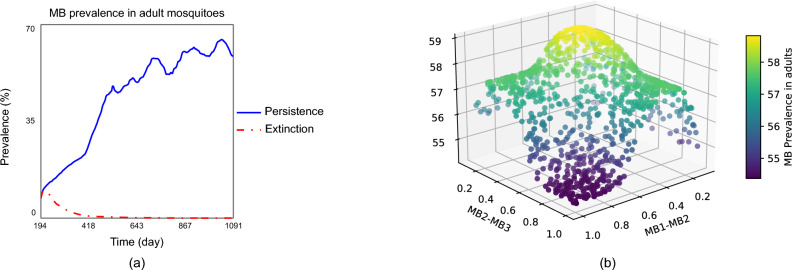
(**a**) and (**b**) illustrate complementary aspects of the effect of the distribution of *MB*-positive mosquitoes across different *MB* intensity levels. (**a**) Scenario of persistence and extinction of *MB*-positive mosquitoes, associated with the distribution of the *MB* intensity. (**b**) Variation of the prevalence of *MB*-positive mosquitoes in adults with the MB progress rates (*MB1-MB2*) and (*MB2* to *MB3*).

Figure 2a depicts two potential evolution scenarios for *MB* prevalence in mosquitoes, related to the progression rates from *MB1* to *MB2* and from *MB2* to *MB3*: persistence and extinction. In this context, progression refers to an increase in *MB* intensity within the lifetime of an infected mosquito.

These outcomes are determined by the reference *MB* progression rates shown in Table 1.

**Table 1:** Parameters for initialization.

	Reference *MB* progress rate			
Persistence scenario	MB0	MB1	MB2	MB3
	0	0.3	0.2	0
Extinction scenario	MB0	MB1	MB2	MB3
	0	0.01	0.01	0

In the extinction scenario, which might seem unexpected, the progression rates from *MB1* to *MB2* and from *MB2* to *MB3* are very low. Consequently, most *MB*-positive mosquitoes maintain a low *MB* intensity, which is lost from one generation to the next, leading to the extinction of *MB*-positive mosquitoes. Figure 2b illustrates the variation in the prevalence of *MB*-positive mosquitoes with different progression rates of *MB* intensity in the mosquitoes. The curve in Figure 2b is quite symmetric, indicating that the prevalence increases similarly as the progression rates from *MB1* to *MB2* and from *MB2* to *MB3* increase. This means that the *MB* equilibrium prevalence depends on the overall progress rate, but not highly on the exact estimation of the two progress rates.

### Effect of vertical and horizontal transmission rates

**Fig. 3:**
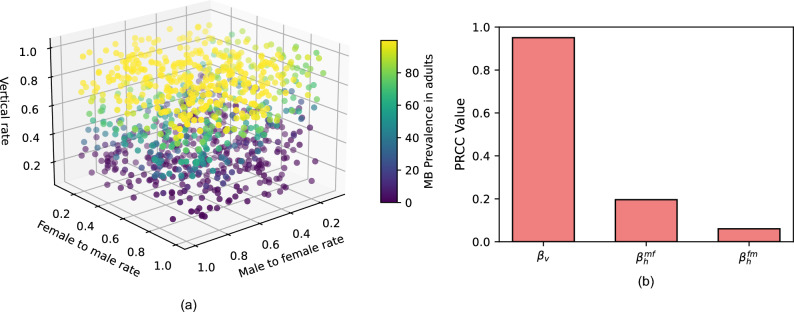
(**a**) and (**b**) illustrate the impact and sensitivity of the prevalence of *MB*-positive mosquitoes to variations in vertical and horizontal transmission rates. (**a**) Variation in prevalence of *MB*-positive mosquitoes with vertical, male-to-female, and female-to-male transmission rates. (**b**) Partial Rank Correlation Coefficients of *MB*-positive mosquito prevalence with respect to the transmission rates.

Figure 3a displays the variation of the *MB* prevalence with the vertical transmission, male-to-female and female-to-male horizontal transmission rates.

From Figure 3a, we observe that in general, a low *MB*-prevalence in adults is associated with a low vertical transmission rate. In addition, a high vertical transmission rate that is associated with a low *MB*-prevalence in adults, is also associated with a low male-to-female horizontal transmission rate. In addition, Figure 3b illustrates the PRCC (Partial Rank Correlation Coefficient) values for each of the transmission rates: vertical transmission ($\beta _{v}$), male-to-female horizontal transmission rate ($\beta _{h}^{mf}$) and the female-to-male horizontal transmission rate ($\beta _{h}^{fm}$). We observe that vertical transmission has the highest impact, followed by the male-to-female horizontal transmission rate. In contrast, the female-to-male has a low effect on the prevalence of *MB*-positive mosquitoes.

### Effect of climatic variables

**Fig. 4:**
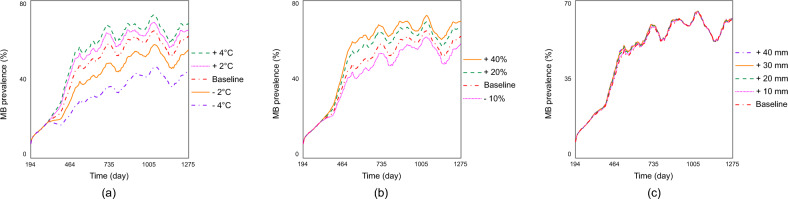
(**a**), (**b**), and (**c**) show the effect of environmental variations (temperature, humidity, and rainfall) on the prevalence of *MB*-positive adult mosquitoes. (**a**) Under temperature adjustments: +2°C, +4°C, –2°C, and –4°C. (**b**) Under humidity adjustments: +20%, +40%, –10%. (**c**) Under rainfall adjustments: +10mm, +20mm, +30mm, +40mm.

From the sensitivity analysis, it is clear that variations in climatic variables significantly affect the spread of *Microsporidia MB*. Temperature emerges as the most influencing factor, followed by rainfall and humidity. By comparing the *MB* prevalence in adult mosquitoes corresponding to various adjustments in the initial temperature data ranges ($+ 2^{\circ }\text {C}$, $+ 4^{\circ }\text {C}$, $- 2^{\circ }\text {C}$ and $- 4^{\circ }\text {C}$), we observe that the prevalence is higher in hotter climates. The baseline temperature datasets range from $21.25^{\circ }\text {C}$ to $30^{\circ }\text {C}$. Adjusting the temperature by $+ 2^{\circ }\text {C}$, $+ 4^{\circ }\text {C}$, $- 2^{\circ }\text {C}$, $- 4^{\circ }\text {C}$, shifts this range to [$23.25^{\circ }\text {C}$ - $32^{\circ }\text {C}$], [$25.25^{\circ }\text {C}$ - $34^{\circ }\text {C}$], [$19.25^{\circ }\text {C}$ - $28^{\circ }\text {C}$] and [$17.25^{\circ }\text {C}$ - $26^{\circ }\text {C}$], respectively. Results (Figure 4a) suggest that *MB* spread more effectively in hotter climates.

The impact of humidity was also assessed by adjusting the initial dataset, which ranged from 12% to 100%, with shifts of (−10%, +20% and +40%) while maintaining the maximum humidity at 100%. Results are depicted in Figure 4b. Figure 4b shows that *MB* spread performs better in higher humidity settings. Regarding the effect of rainfall, no significant changes were observed when the initial rainfall datasets were adjusted by (+10mm/day, +20mm/day, +30mm/day and +40mm/day), as shown in Figure 4c.

## Discussion

We developed a System Dynamics model to investigate the temporal dynamics of *MB*-positive mosquitoes under environmental variability. The model analyses the interactive dynamics of humans, mosquitoes and the endosymbiont *Microsporidia MB* under specific assumptions. The mosquito population is structured according to immature stages, age structure, gender distribution, mating status, the *Plasmodium* infection stages and the *MB*-intensity classes, while environmental drivers such as temperature, humidity, and rainfall are incorporated as external inputs. Key assumptions include neglecting immunity in the human population, time-based progression rate between *MB* intensity classes, and unique mating occurrence for females. The model also allows for flexible representation of release strategies, defined by release timing, total duration, number, and the characteristics of released mosquitoes, including age, sex, mating status, *MB*-infection and *Plasmodium*-infection. In addition, established interventions such as Indoor Residual Spray (IRS), Insecticide-Treated Nets (ITNs) and Environmental Management Measures (EMG) are included to explore the potential of integrated malaria control approaches.

The analysis provides insights into the dynamics of mosquito populations infected with *Microsporidia MB* and their implications for malaria control. Results are derived from simulations calibrated against field data on mosquito abundance and *MB* prevalence, and further explored through sensitivity analyses focusing on *MB* intensity, transmission pathways, and environmental variability. Model outputs show good agreement with the observed temporal patterns, capturing the underlying trends in both mosquito abundance and *MB* prevalence. Although short-term fluctuations are not fully reproduced, the low bias between simulated and observed values indicated that the model provides a consistent representation of the system’s overall behaviour, supporting its use as a baseline for evaluating alternative intervention scenarios.

The findings highlight several mechanisms that are likely to influence the establishment and persistence of *MB* in mosquito populations. Vertical transmission emerges as the most influential pathway, followed by male-to-female horizontal transmission, while female-to-male transmission plays a comparatively limited role. These transmission modes act jointly to shape *MB* dynamics within the population: vertical transmission supports the persistence of infection across generations, whereas horizontal transmission, particularly from males to females, facilitates its propagation, especially when prevalence is low. The relatively weak contribution of female-to-male transmission reflects its indirect effect, as newly infected males contribute to transmission only through subsequent mating events with uninfected females. This result corroborates with the previous work analyzing the interactive dynamics of *MB*-positive mosquitoes^25^. Taken together, these results suggest that the effectiveness of release strategies depends on the combined performance of both vertical and horizontal transmission processes.

In this context, transmission efficiency emerges as a central determinant of intervention success. Given that transmission rates are intrinsic to a given *MB* strain, the sensitivity of model outcomes to these parameters indicates that variation in transmission efficiency could substantially influence intervention performance. Improving transmission fidelity could increase the likelihood of stable establishment following release, suggesting that future work may benefit from exploring variation in *MB* transmission performance across strains or environmental conditions. Similar considerations have been central to the deployment of *Wolbachia*-based strategies, where differences in transmission efficiency and fitness effects have strongly influenced establishment outcomes^40,41^. Any such approaches would require careful evaluation to ensure stability, safety, and consistent transmission-blocking performance under field conditions.

The distribution of *MB* intensity within infected mosquitoes also plays a critical role. Simulations indicate that populations dominated by low-intensity infections tend to lose infection over successive generations, whereas higher intensity distributions are associated with sustained persistence. This behaviour reflects the representation of intergenerational dynamics in the model, whereby *MB* intensity may decrease from parent to offspring, limiting effective transmission when infections are predominantly low-intensity. Within this context, the results suggest that the effectiveness of release programs may depend not only on the number of mosquitoes released but also on the infection profile of those individuals, highlighting the importance of maintaining adequate *MB* intensity during rearing and release.

Environmental conditions further influence these dynamics. Variations in temperature and humidity are associated with changes in *MB* prevalence in the model, with warmer and more humid conditions generally favouring higher levels of infection. While these patterns are consistent with broader ecological understanding of mosquito systems, they arise from the representation of environmental effects on *MB* transmission and progression within the modelling framework. As such, the direction of these relationships is suggestive of potential trends, whereas their magnitude and functional form remain dependent on model assumptions and would benefit from further empirical validation.

Several assumptions underlying the model define its scope and should be considered when interpreting these results. In particular, the assumption of homogeneous mixing and spatial distribution of *MB*-positive and *MB*-negative mosquitoes simplifies mating interactions and may overestimate contact rates compared to natural settings, where spatial structure and behavioural heterogeneity can limit encounters. As a result, the spread of *MB* may be slower or more localized than predicted, potentially reducing the effectiveness of horizontal transmission and release strategies. The model further assumes a single strain of *Microsporidia MB* and focuses on one vector species, reflecting current observations from Kenya; however, the presence of multiple strains and coexisting vector species could influence transmission dynamics, particularly for horizontal transmission pathways. In addition, climate-driven effects on transmission rates were incorporated based on assumptions. While similar patterns have been observed for *Wolbachia*^42^, these relationships remain to be empirically validated for *Microsporidia MB*, and their strength and functional form may differ under field conditions.

These limitations highlight the importance of validation under more realistic ecological conditions. Semi-field and field studies incorporating spatial heterogeneity, mating behaviour, environmental variability, and species composition would provide critical evidence to assess how *MB* spreads following release and to refine model assumptions. Incorporating spatial structure into future modelling frameworks would further improve the representation of transmission dynamics and enhance predictive accuracy. Future work should also focus on linking model predictions to epidemiological outcomes by integrating real-time data on malaria prevalence across multiple regions, including Ahero and other settings where data are available. In parallel, assessing the operational costs associated with rearing and releasing *MB*-positive mosquitoes, and identifying target areas where this strategy could achieve the greatest impact, will be essential for translating modelling insights into practical interventions. Overall, this study provides a mechanistic framework for understanding the factors that influence the spread and persistence of *Microsporidia MB* in mosquito populations and offers guidance for the design and optimization of *MB*-based malaria control strategies, while highlighting key areas where further empirical evidence is needed.

## Methods

### Data sources

The System Dynamics model was calibrated to reproduce the trends observed in the Ahero region. Model parameters were informed by a combination of peer-reviewed literature, official datasets from international organizations (e.g., WHO malaria reports), and field data collected in Ahero, Kenya. Field data were used to calibrate mosquito population dynamics and the prevalence of *Microsporidia MB* (*MB*) in adult mosquitoes.

Between July 2021 and December 2023, adult mosquitoes were collected in the Ahero region using manual mouth aspirators. Collections targeted indoor resting mosquitoes within households. Each sampling round involved between four and twelve households (mean $\approx$ 10 households), and sampling occurred at irregular intervals with an average frequency of approximately two days (range 1–33 days). Mosquitoes were identified morphologically as members of the *Anopheles gambiae* species complex, predominantly *Anopheles arabiensis*.

The collected dataset included adult mosquito abundance, climatic variables (rainfall, humidity, and temperature), the number of oviposited mosquitoes, the number of *MB*-positive mosquitoes among oviposited mosquitoes, and the prevalence of *MB*-positive mosquitoes. Adult mosquito abundance represents the number of mosquitoes collected during household sampling and is interpreted as an index of indoor resting adult mosquito density, rather than a direct estimate of the total mosquito population size in the study area.

Climatic variables were recorded on-site during mosquito sampling campaigns at a local weather station operated by the National Irrigation Board at the *Ahero* irrigation scheme, and therefore represent the environmental conditions experienced by the mosquito populations during the study period. NDVI data were derived from Sentinel-2 satellite imagery at a spatial resolution of 10 m, extracted over the study area, and matched to the corresponding mosquito collection dates to represent vegetation dynamics influencing mosquito habitats. After data cleaning to remove inconsistent or incomplete records, the climatic time series were incorporated into the System Dynamics model as exogenous inputs through data input files in the Stella Architect modelling platform. Lagged effects were considered to account for delayed impacts of environmental conditions on mosquito development and population dynamics.

Oviposited mosquitoes refer to adult female mosquitoes that successfully laid eggs at least once after collection under laboratory conditions. These females were retained for subsequent screening of *MB* infection status. *MB* infection was detected using molecular assays based on PCR amplification of the *Microsporidia MB* 18S rRNA gene. The prevalence of *MB*-positive mosquitoes was calculated as the proportion of PCR-positive individuals among the screened oviposited mosquitoes.

To simulate the dynamics under study, a broad range of parameters was specified for mosquitoes, humans, and the environment. Some parameters are general (see Table 2 constant parameters, Table 3 for arrays, and Table 4 for graphical functions). Others are location-specific, such as climatic variables and their impact on population dynamics and are calibrated to the collected data (see Tables 5 and 6). Additionally, parameters were defined to initialize the model (see Table 7) and parameters that define the release policy (see Table 8). Model parameters originate from three main sources: literature-based values reported in the corresponding tables and treated as fixed parameters; parameters whose values were assumed when direct estimates were not available, while remaining consistent with available data or biological knowledge; and parameters estimated through calibration to field data collected in Ahero. The calibration procedure and parameter ranges are described in the Model Calibration section. These data include:

- *MB* transmission rates and environmental effects: paternal, vertical, and horizontal transmission rates, attractiveness of *MB*-positive mosquitoes.
- Mosquito lifecycle and environmental effects: development rates and survival in immature stages, sex ratio, *Plasmodium* development rates, mating rates, biting rate and mosquito infectivity.
- Malaria and human population dynamics: human population, human infectivity, recovery rate.
- Release policy: release number, release stop time, release duration, policy start time.
- Effectiveness, and coverage of additional intervention strategies (IRS, ITN, and EMG).

In the next subsection, we provide an overview of the modelling process.

### Modelling procedure

The model is designed to support the analysis of how targeted *Microsporidia MB* release program can contribute to reducing malaria case burden, based on its transmission-blocking potential. In this work, we apply the system dynamics (SD) modelling approach to investigate malaria control through vector population replacement. SD modelling allows the explicit representation of key mechanisms governing malaria transmission, including feedback loops between mosquito population dynamics and malaria infection prevalence, time delays associated with mosquito development stages, and the influence of external drivers such as climate conditions. This approach enables the simulation of alternative intervention scenarios and the evaluation of their long-term effects on mosquito populations and malaria transmission. By capturing these dynamic interactions, the model can be used to assess potential outcomes, benefits, and risks of vector replacement strategies.

The modelling process involves the following steps:

1. Model conceptualization: This step involves reviewing available information on mosquito, malaria, and *MB* dynamics and defining the key assumptions of the system. A system dynamics model is constructed in Stella Architect by defining stocks (e.g., mosquito population compartments), flows (e.g., birth, infection, and mortality rates), and converters representing environmental or epidemiological drivers. These components structure the feedback relationships and temporal dynamics within the model.
2. Model calibration: This step involves defining the mathematical relationships governing the model structure. Model parameters are calibrated using available empirical data and validated against reported malaria and mosquito population data before conducting simulations.
3. Sensitivity analysis: A sensitivity analysis is conducted to evaluate the influence of key parameters on model outcomes. This analysis helps identify the variables that most strongly affect the dynamics of *MB*-positive mosquitoes and malaria transmission.

In the following sections, we provide a detailed description of the modelling steps.

### Model conceptualisation: model structure and global assumptions

The model’s structure focuses on representing the dynamic interaction between humans, *Anopheles*, *Plasmodium* parasites and *Microsporidia MB*. Environmental factors affect the intensity of those interactions and change over time to exogenously mimic the fundamental conditions observed in a specific area.

**Fig. 5:**
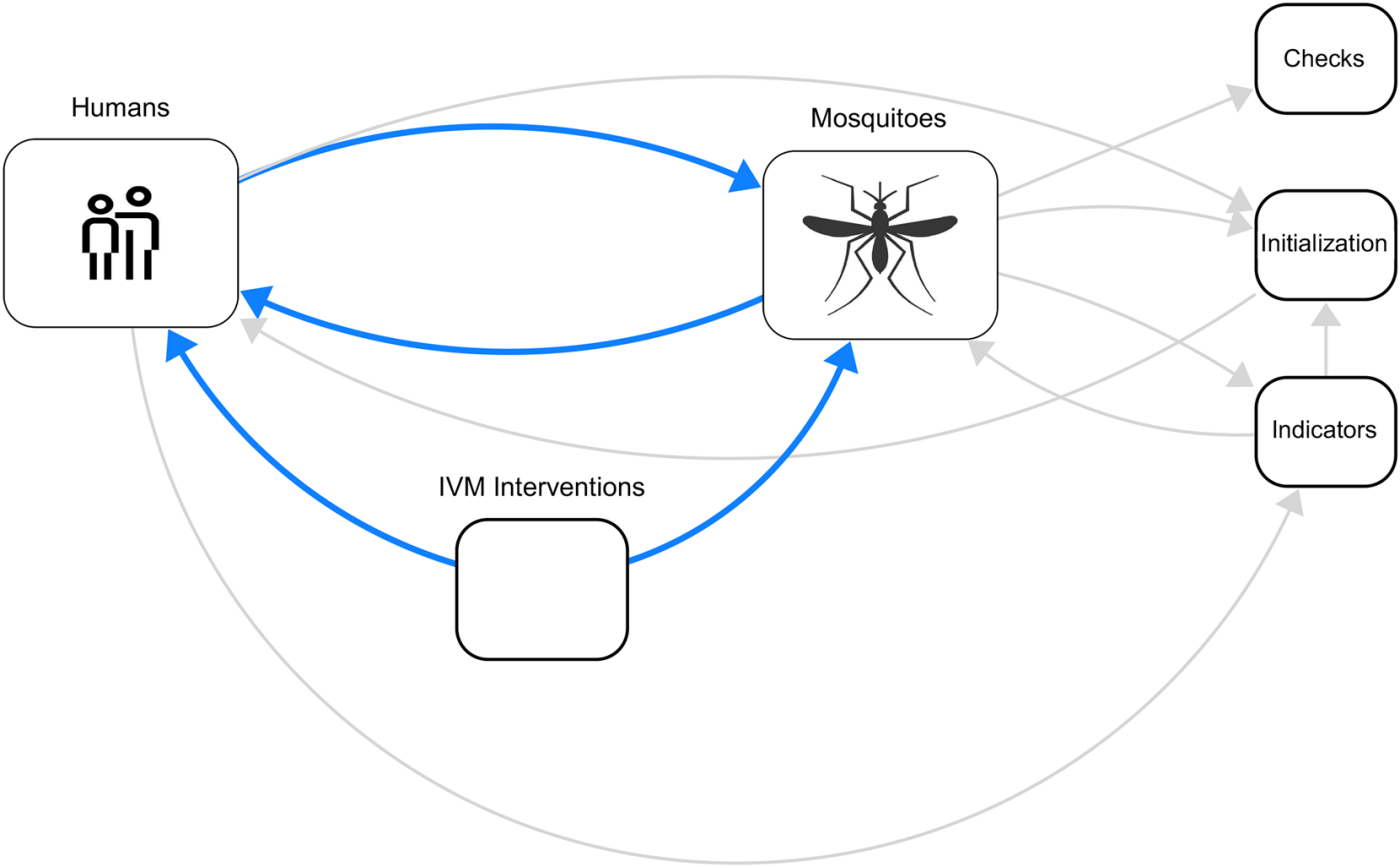
General overview of the System Dynamics model.

The model is developed according to the malaria parasite *Plasmodium* life cycle. Malaria infection involves both the human host and the vector host. In fact, the transmission of the *Plasmodium* from the mosquito to the human or from the human to the mosquito happens during the mosquito blood-feeding. Thus, the human population, mosquito population, and the parasite transmission success probability between a mosquito and a human determines the malaria disease progress. The model includes three primary sectors: Mosquitoes; Humans; and integrated vector management (IVM) policies. Within the Mosquitoes sector, we track the life cycle of mosquitoes, and of the *Plasmodium* parasite and *MB* inside the mosquito. These dynamics are driven by endogenous mechanisms (e.g., reproduction feedback loop) and are affected by external factors (e.g., weather). The Humans sector represents the process of malaria infection and recovery, under the assumption of constant population. Considering the evidence of the need to adopt IVM for malaria control^43^, we design a third module for IVM interventions that consider common malaria interventions, such as the use of insecticide-treated nets (ITNs), Indoor Residual Spray (IRS) and Environmental Management Measures (EMG). Additionally, the model includes three supporting sectors (Checks, Indicators and Initialisation), which provide useful consistency checks, indicators, and initialisation values. The global overview of the model is shown in Figure 5, and the description of the main sectors is provided below.

We consider a historical period of about one year and a future time horizon of two to three years. Time constants are expressed in days, and we are using a timestep of one day. We adopt an extended time horizon to allow for the identification of long-term consequences of a policy, beyond their most immediate short-term impacts, e.g., malaria control can reduce a population’s immunity in the long run and thus increase susceptibility to a future epidemic.

#### Mosquito sector

**Fig. 6:**
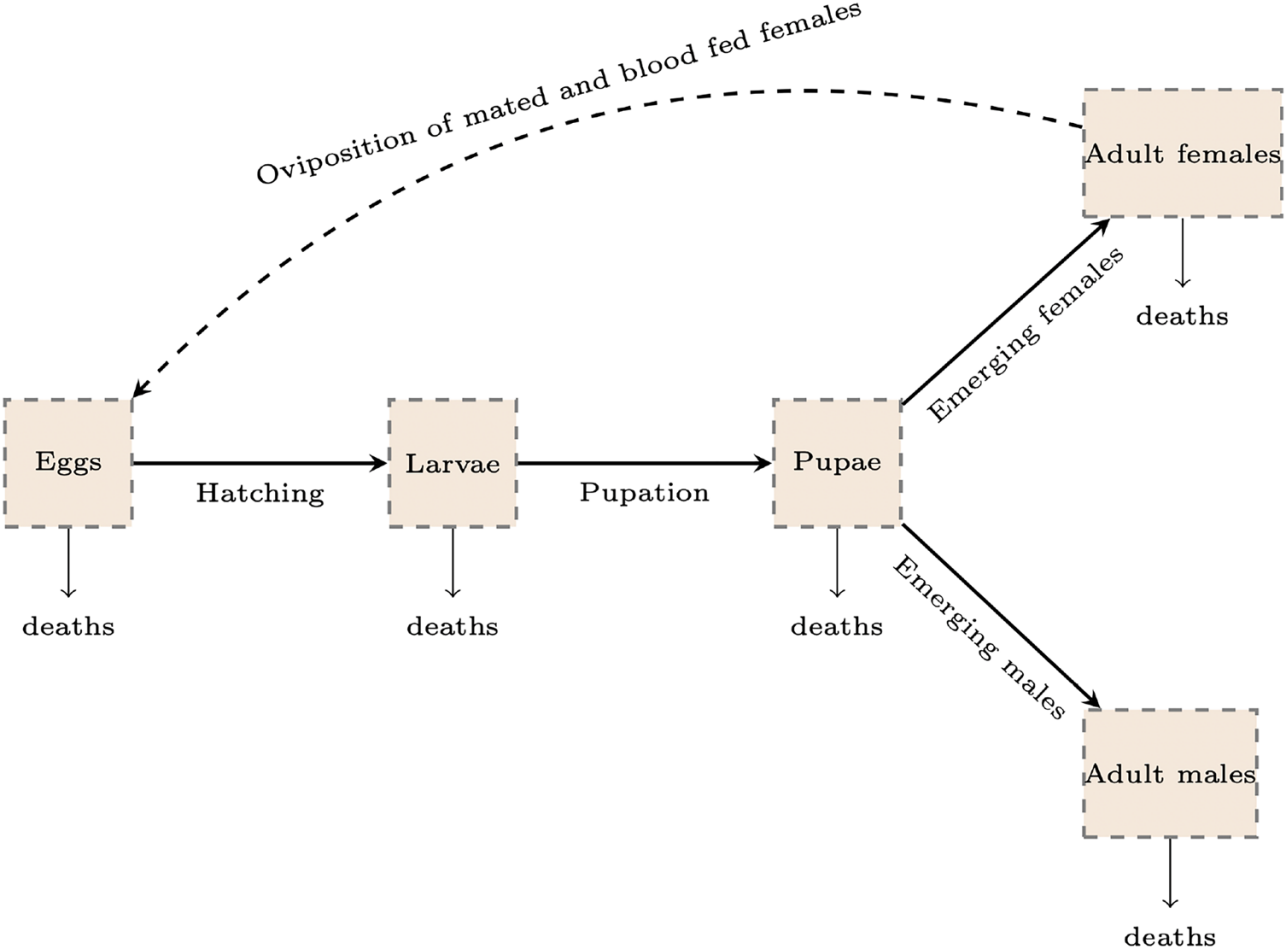
Mosquito life cycle: from immature to adult stage, as described in^44^.

The mosquito sector represents the development of the mosquito population over time, including its distribution by age, sex, mating status, *MB*-infection and *Plasmodium* infection. To achieve an accurate estimation of the adult population, we track mosquito development from the immature stages to the adult stage, as depicted in Figure 6.

According to the mosquito lifecycle^44^, newly emerged adult females mate, and blood feed. Although it is not clear which event occurs first^45^, we assume that females mate first and then blood-feed. After blood-feeding, fertile females lay eggs that will successively develop into larvae, pupae, and adults, depending on the survival and development rates. The survival and development rates in the immature stage depend on time as well as environmental conditions such as temperature. Besides tracking the progression of the mosquitoes through their life stages, a significant challenge is to define how the distribution of the mosquitoes evolves with respect to age, sex, mating status, *MB*-infection status, and *Plasmodium* status. The main assumptions are described below.

**Fig. 7:**
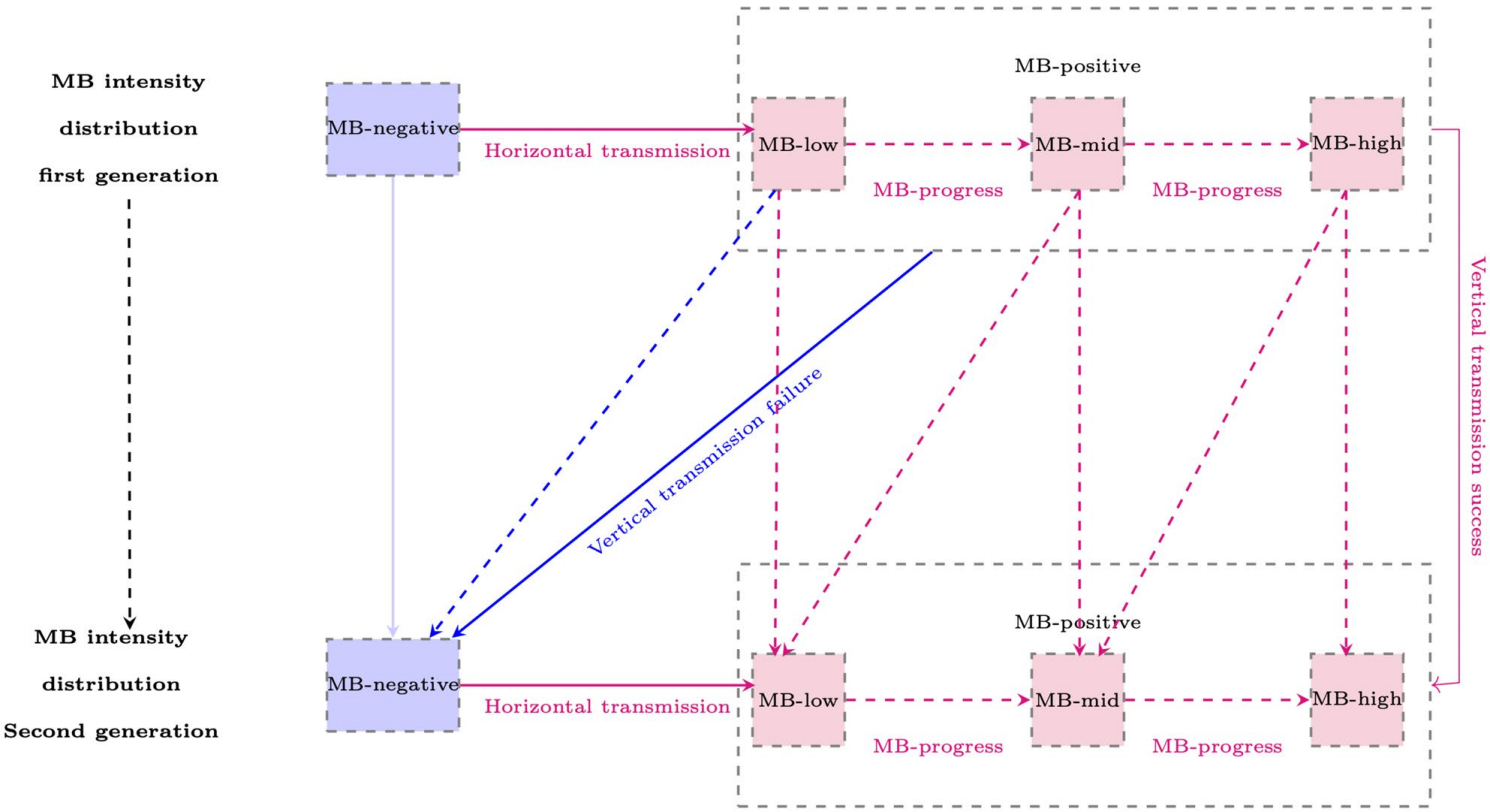
*Microsporidia MB* transmission from one generation to the next as described in^20,24^ with additional assumptions in the model formulation.

1. **Age and sex**. Adult mosquitoes (both females and males) have an age range from 0 to 40 days^44^, with progress being time-based. The sex distribution is not considered during the immature stages. The number of emerging adult females or males after the pupae stage is defined based on a constant sex ratio.
2. **Mating**. Mating status is categorized as either unmated or mated, specifically to consider a unique mating occurrence^46^ for female mosquitoes. Since males can mate multiple times, their mating status is not tracked, and they are all considered unmated. The number of females mating per unit of time is based on the new emerging females, the average time for mating after emergence, and the average number of female mosquitoes that mate per age per unit of time.
3. ***MB intensity***. We characterized four levels of *MB* intensity: negative, low (MB1), mid (MB2), and high (MB3), representing increasing levels of symbiont load within the host. These categories are based on qPCR-derived measures of *MB* abundance (normalized MB18S/S7 ratios) reported in the literature^47^ and provide a discretized representation of continuous variation in infection intensity. The “negative” class corresponds to mosquitoes in which *MB* is not detected. The model does not explicitly account for differences in diagnostic detectability across *MB* intensity levels. In particular, low-intensity infections may be more likely to result in false negatives in molecular assays (e.g., PCR-based detection). However, due to the absence of quantitative data linking *MB* intensity levels to detection thresholds, we assume that all *MB*-positive mosquitoes are detected with equal probability. Under this assumption, the observed *MB* prevalence is treated as a proxy for the true prevalence, and potential under-detection at low intensity is not explicitly modelled. Then, the *MB*-intensity variability across generations (resumed in Figure 7) is affected by the time, seasonality, vertical transmission, and horizontal transmission infection success.

First, an *MB*-negative mosquito (either male or female) can acquire the *MB* infection after mating with an *MB*-positive partner, depending on the horizontal transmission success rate^20,24^. This horizontal transmission efficiency from an *MB*-positive male to an *MB*-negative female or from an *MB*-positive female to an *MB*-negative male increases with the *MB* intensity level in the *MB*-positive partner. Newly horizontally infected mosquitoes are assumed to enter the lowest *MB* intensity class. This assumption is consistent with observations that *MB* density increases over time within infected mosquitoes, and reflects a simplified representation in the absence of detailed data on the initial distribution of infection intensity following horizontal transmission. Secondly, we assume a time-based natural increase of *MB* intensity in mosquitoes. Over time, a proportion of mosquitoes move from low to mid-intensity and from mid to high intensity. This *MB* intensity progression rate is influenced by the temperature and humidity. Thirdly, *MB*-negative fertile females will always lay *MB*-negative offspring. On the other hand, *MB*-positive fertile females can transfer the *MB* infection to their offspring (vertical transmission). According to the experimental studies^20,24^, the vertical transmission success rate varies from 0.45 to 1, allowing for either perfect or imperfect vertical transmission. In the case of imperfect vertical transmission, a proportion of the offspring, determined by vertical transmission failure rate, will be *MB*-negative. The vertical transmission rate is increased with the mother *MB* level and is temperature, humidity and NDVI (Normalized Difference Vegetation Index) dependent. Additionally, there is a decrease in *MB* intensity in the offspring compared to the mother. For instance, a fertile female with low *MB* intensity can produce both *MB*-negative offspring and offspring with low *MB* intensity. Finally, for adaptability in the model, we hypothesize a situation where newly horizontally infected mosquitoes do not initially have the *Plasmodium*-blocking ability, but the next generation does. In this context, newly horizontally infected mosquitoes are not counted as *MB*-positive. Instead, we define a paternal-mediated vertical transmission rate, representing the proportion of eggs that acquire the *MB* infection among those laid by females that were newly infected through horizontal transmission (i.e., through mating). Offspring infected through this pathway are assumed to have a low *MB* intensity, consistent with the assumption for newly horizontally infected mosquitoes. An important modelling assumption is that *MB* infection status and intensity are conserved from the egg stage to the adult stage. This assumption is supported by the absence of detailed empirical data quantifying within-host loss or dilution of *MB* during mosquito development.

In the model implementation, transmission processes were formulated using explicit intensity-dependent and environment-dependent functions. The vertical transmission rate $\beta _v$ was defined as a weighted combination of a reference transmission rate and an intensity-dependent component, bounded between 0 and 1:

$$\beta _v = \min \left( 1,\ \beta _0 \left[ (1-\alpha ) + \alpha \cdot f(I)\right] \cdot g(E)\right) ;$$

where $\beta _0$ is the reference vertical transmission rate, $\alpha$ is the intensity scaling parameter (set to 0.5), and *f*(*I*) is a normalized linear function of the *MB* intensity level *I*:

$$f(I) = \frac{I - 1}{I_{\max }/2}$$

This formulation captures the increase in transmission efficiency with increasing *MB* intensity, consistent with experimental observations^20,24^.

Environmental effects were incorporated multiplicatively using elasticity-based functions:

$$g(E) = \left( \frac{T}{T_0}\right) ^{\epsilon _T} \left( \frac{NDVI}{{NDVI}_0}\right) ^{\epsilon _N} \left( \frac{H}{H_0}\right) ^{\epsilon _H}$$

where *T*, *NDVI*, and *H* denote temperature, vegetation index, and relative humidity, respectively, and $T_0$, ${NDVI}_0$, and $H_0$ are baseline values. The elasticity parameters define the sensitivity of *MB* transmission rates to environmental variation, and were estimated during model calibration, reflecting known ecological effects on mosquito and microbial dynamics.

Similarly, horizontal transmission was modelled as an intensity-dependent process. The horizontal transmission rate $\beta _h$ was defined as:

$$\beta _h = \beta _{h0} \left[ (1-\alpha ) + \alpha \cdot f(I)\right]$$

where $\beta _{h0}$ is the reference horizontal transmission rate and $\alpha$ is the intensity scaling parameter (set to 0.5).

To address potential identifiability and over-parameterization issues arising from the combined effects of *MB* intensity and multiple environmental drivers (temperature, humidity, and NDVI), the model was designed using simple and consistent functional forms. The effect of *MB* intensity is represented through a single normalized linear function and a fixed scaling parameter, while environmental influences are incorporated through multiplicative elasticity terms. This approach limits the number of free parameters and avoids introducing separate nonlinear relationships for each driver, thereby reducing the risk of compensatory effects between parameters. In addition, only a subset of parameters, including the environmental elasticities, was estimated through calibration, while others were fixed based on literature or biological assumptions.

**Fig. 8:**
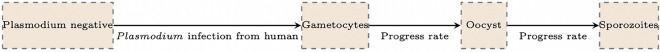
Development stages of the *Plasmodium* in the mosquito.

4. ***Plasmodium infection***. We consider four successive stages in the *Plasmodium* infection, which are: *Plasmodium* negative, *gametocytes*, *oocyst* and *sporozoites* (Figure 8).

Mosquitoes infected with *MB* (at low, mid, or high) intensity have the transmission-blocking property. Experimental studies^20,24^, show that *Plasmodium* development is hindered at the *oocyst* stage in *MB*-positive mosquitoes, impairing the transmission of the *Plasmodium* to humans. In this model, we assume that the transmission-blocking is influenced by the *MB* intensity in the mosquito. Mosquitoes with low *MB* intensity have imperfect transmission blocking, while those with mid or high *MB* intensity exhibit perfect transmission blocking. After a mosquito gets *Plasmodium* infection, it progresses through the *gametocyte* and *oocyst* stages before maturing into *sporozoites*^48^. The time between mosquito infection and maturation into sporozoites is known as the extrinsic incubation period, which is temperature-dependent. We define the extrinsic incubation time in terms of the proportion of time required for gametocytes to progress into *oocysts* and for *oocysts* to progress into *sporozoites*.

One of the main objectives of our study is to analyze the effectiveness of vector population replacement by releasing *MB*-positive mosquitoes for malaria control. To achieve this, we include an additional inflow of *MB*-positive mosquitoes into the existing population, thus altering the existing prevalence of *MB* in adult mosquitoes. The number of released mosquitoes can be set and classified by age, sex, mating status, *MB*-infection and *Plasmodium*-infection to reflect different release strategies. The release parameters include:

- the release’s start and end time, representing the specific timing for the beginning and end of mosquitoes’ release,
- specific months of release, which defines the months the releases will occur,
- frequency of releases: within each month, mosquitoes can be released over one or more days to reflect the varying levels of *MB*-positive mosquitoes’ availability.

This extensive set of parameters allows us to test a broad range of alternative release strategies.

#### Human sector

The human sector estimates the number of infected humans per unit of time. Overall, the model structure is similar to an SIS compartmental model. We maintain a low level of detail and use a series of simplifying assumptions for this sector to allow the model’s application to locations without detailed demographic and epidemiological data. First, we assume a constant human population divided into susceptible and infectious individuals. This assumption simplifies the representation of the human component of the system and allows the analysis to focus on mosquito population dynamics and the spread of *Microsporidia MB*. Over the relatively short simulation horizon considered in this study (approximately two to three years), demographic changes such as births, deaths, and migration are expected to have a limited influence on malaria transmission dynamics. Therefore, the total human population size is treated as constant, while individuals transition between susceptible and infectious states. Susceptible humans move to the stock of infected humans after acquiring the *Plasmodium* infection. The infection rate is expressed as the product of the number of infectious bites a susceptible individual is exposed to (also called Entomological Inoculation Rate (EIR)) and the mosquito infectivity, which is the *Plasmodium* transmission efficiency through an infectious bite on a susceptible human. The variables involved in the formulation of the mosquito infectivity and the EIR are illustrated in the human module. The infection rate is reduced by the effect of the use of Insecticide-treated bed nets. Secondly, we consider that infected humans are immediately infectious, i.e., we neglect the *Plasmodium* incubation period. This simplification is justified by the short time delay, which does not significantly affect the overall duration of the infectious phase, which can last up to three years^49^. After infection, there is an average recovery time, after which the infected humans recover, and return to the susceptible state. Recovery here refers to the complete elimination of the parasite from the human body, not just the disappearance of the symptoms. By this formulation, we implicitly assume that as long as humans are in the recovery stage, they are immune to further reinfection.

#### IVM interventions, checks, initialisation, and indicators

Among the Integrated Vector Management (IVM) strategies that have been implemented for malaria control, we distinguish the use of Insecticide Treated Nets (ITNs), Indoor Residual Spray (IRS) and Environmental Management Measures (EMG). ITNs protect humans against mosquito bites and thus, reduce malaria incidence. IRS increases mosquitoes mortality by applying insecticides on the walls or surfaces of houses. EMG reduces the larvae carrying capacity. As these methods are insecticide-based, the duration of their effectiveness is limited. Therefore, the impact of these IVM interventions on malaria reduction depends on the coverage, their effectiveness, and the duration of effectiveness. The IVM interventions module aims to express the coverage of each intervention. These coverages, considered as stocks, are increased at a rate depending on the new implementations and the time required for implementing those interventions. They are decreased at a rate depending on the duration of effectiveness of the intervention. Once, the coverage for each intervention is expressed, the effect on malaria incidence is determined by the product of the coverage and the effectiveness.

The checks module ensured the accurate distribution of the mosquito population by *MB* intensity. Verification is done at three stages: The adult population is distributed such that the sum of the proportion of the adult mosquitoes in each *MB*-intensity group is equal to 1. It was ensured that the total oviposition number matches the sum of total oviposition per mother *MB* intensity. It was further ensured that the number of *MB*-positive offspring is conserved while expressing the decrease in the *MB* intensity during vertical transmission. This involves verifying the redistribution of the offspring’s *MB* intensity relative to the mother’s *MB* intensity.

Defining initial values is essential in model development as it determines the behaviour of the system. The initialization sector provides initial values for the main sector of mosquitoes and humans. This sector describes the initial distribution of the mosquito population with respect to age, sex, mating, *MB*-infection, and *Plasmodium* infection. The module also provides the initial number of infected humans.

Finally, the module of indicators highlights the key variables of the model which are: the human or malaria prevalence, the *Plasmodium* prevalence and the *MB*-prevalence in adult mosquitoes.

### Model calibration

The calibration process involves a comprehensive dataset collected between July 13, 2021, and December 27, 2023, and the average time between collection is 2 days (from 1 to 33 days). This period is equivalent to days 194 through 1091 in the model with July 1, 2021, being the first day. It was observed that the datasets present two baselines for the prevalence of *MB*-positive mosquitoes. The first spans from July 13, 2021, to October 12, 2022, and the second from October 17, 2022, to December 27, 2023. The first baseline is used to calibrate the model, and the second to observe the effects of an increased prevalence of *MB*-positive mosquitoes on malaria incidence. Each collection involved an average of 10 households (ranging from four to twelve households). Thus, we are capturing the mosquito dynamics over 10 households. The number of total mosquitoes used for the model calibration was obtained by weighting the collected mosquitoes to 10 households using the formula:

1 $$\begin{aligned} \dfrac{\text {Number \ of \ collected \ mosquitoes}}{\text {Number \ of \ households}} \times 10. \end{aligned}$$

To ensure consistency, we consider the number of humans over 10 households, with an average of 6 humans per household. For the calibration, we assume that the prevalence of *MB*-positive mosquitoes amongst the oviposited mosquitoes (given in the data) is a measure of the prevalence of *MB*-positive mosquitoes in the adult mosquito abundance. The calibration is done both for the adult mosquito abundance and the *MB*-prevalence: the adult mosquito abundance is calibrated over the parameters and ranges in Table 5; and the *MB*-prevalence in adult mosquitoes is calibrated over the parameters and ranges in Table 6.

We ran the optimizations using the *Powell* method in the *Stella Architect* software and minimized using the squared error the difference between the data and the simulated variables. The fit of the model to historical data was evaluated using two error metrics:

2 $$\begin{aligned} RMSPE = \sqrt{\dfrac{1}{n}\sum _{t = 1}^{n}\left( \dfrac{y_{t} - \bar{y_{t}}}{y_{t}}\right) ^{2}}; \end{aligned}$$

and the TBIAS (Mean Bias) error given by:

3 $$\begin{aligned} TBIAS = \sqrt{\dfrac{1}{n}\sum _{t = 1}^{n}\left( \bar{y_{t}} - y_{t}\right) }; \end{aligned}$$

where *n* is the number of data, *t* is the time unit, $y_{t}$ represents observed values from the data and $\bar{y_{t}}$ stand for the predicted values generated from the model.

Values of *RMSPE* and *TBIAS* close to zero indicate that the model is closely aligned with real-world data. *RMSPE* (Root Mean Square Percentage Error) assesses accuracy relative to the magnitude of observed values while *TBIAS* (Mean Bias) highlights the average deviation between observed and predicted values. These metrics provide insights into the model’s accuracy.

### Sensitivity analysis

The sensitivity analysis was conducted to determine how key factors, such as the *MB* intensity progression rates (representing increases in *MB* intensity within the lifetime of infected mosquitoes) and the *MB* transmission rates, affect the spread of *MB*-positive mosquitoes. To assess the impact of the progression rates of *MB* intensity in mosquitoes on the prevalence of *MB*-positive mosquitoes, we conducted a sensitivity analysis with 1000 runs. In this analysis, we varied the progression rates from *MB1* to *MB2* and from *MB2* to *MB3*, following a uniform distribution ranging from 0.1 to 1. The resulting dataset, representing the variation in the prevalence of *MB* in adults with the progression rates, was then extracted and visually represented.

To evaluate the effect of *MB* transmission rates, we conducted another sensitivity analysis with 1000 runs. Here, we varied the vertical transmission rates, male-to-female, and female-to-male horizontal transmission rates, also following a uniform distribution between 0.1 and 1. Using this data, we computed the Partial Rank Correlation Coefficient (PRCC) to identify the transmission rate with the highest impact on the equilibrium prevalence of *MB*-positive mosquitoes. The PRCC is a measure used in sensitivity analysis to quantify the relationship between model input parameters and the output of interest while controlling for the effects of other parameters. It is computed using the formula:

4 $$\begin{aligned} PRCC(X_{i}, \ Y) = \dfrac{\textrm{Cov}(R(X_{i}), \ R(Y|X_{i}))}{\sigma (R(X_{i}))\sigma (R(Y|X_{i}))}; \end{aligned}$$

where, $R(X_{i})$ is the rank of $X_{i}$, $R(Y|X_{i})$ is the rank of *Y* conditioned on $X_{i}$, $\textrm{Cov}$ denotes the covariance and $\sigma$ denotes the standard deviation.

To assess the effect of climate variability on the prevalence of *MB*-positive mosquitoes, we perform a sensitivity analysis using 250 runs. The temperature, humidity, and rainfall are adjusted by adding constant offsets, which values are drawn from a uniform distribution within the range of −10$^\circ$C to 10$^\circ$C for temperature, −10% to 40% for humidity, and −40 mm to 40 mm for the rainfall). We ensure that all values, including temperature, rainfall, and humidity, remain positive, with humidity specifically constrained to remain below 100%. Confidence plots are then used to evaluate how variations in these climatic variables influence the spread of *MB*-positive mosquitoes.

## Data Availability

Most data are provided within the manuscript. Additional data are available from the corresponding author upon reasonable request.

## Appendix A: Table of parameters

See Tables 2, 3, 4, 5, 6, 7 and 8.

**Table 2:** General parameters for the model (constants).

Parameters	Values	Unit	Reference
Paternal vertical transmission rate	0.1 (increased by *MB* level)	dmnl	Assumed
*MB* horizontal transmission increase by *MB* level	0.5	dmnl	Assumed
Reference female mating proportion	0.7665	dmnl/day	^50^
*MB*-positive male relative attractiveness	2 [1, 2]	dmnl	Calibrated
*MB* vertical transmission increase by *MB* level	0.5	dmnl	Assumed
Maximum eggs failure rate	1	dmnl/day	Assumed
Immature gender distribution	1:1	dmnl	^51^
Oocyst share of plasmodium development time	0.66	dmnl	^52^
Age cohort duration	1	day	Assumed
Part of day lived before dying	0.5	day	Assumed
Average recovery time	405	day	Assumed
Average number of people per households	6	person/household	Consistent with data
Number of households	10	household	Consistent with data
Bites per gonotrophic cycle	1	bite/mosquito	Calibrated

**Table 3:** General parameters for the model (Arrays).

Parameters	Values	Unit	Reference
Reference *MB* progress rate		dmnl/day	Consistent with definition and data
Reference *MB* intensity decrease in vertical transmission rate		dmnl	Consistent with definition and data
Paternal vertical transmission rate distribution by MB level		dmnl	Consistent with definition and data
Threshold low temperature half enzyme inactivation		K	^53^
Threshold high temperature half enzyme inactivation		K	^53^
Enthalpy change with high temperature inactivation		cal/mol	^53^
Enthalpy of reaction activation		cal/mol	^53^
Enthalpy change with low temperature inactivation		cal/mol	^53^

**Table 4:** Parameters defined as graphical functions.

Parameters	Values	Unit	Reference
Egg to adult development timetable.	[0, 31] (Temperature dependent)	days	^54^
Immature phase survival rate table	[0, 0.72] (Temperature dependent)	dmnl	^54^
Indicated mosquito mortality distribution by age	[0, 1] (Increasing with age)	dmnl	^54^
Female mosquito life expectancy table	Temperature dependent	dmnl	^44^
Male mosquito life expectancy table	Temperature dependent	dmnl	^44^
Extrinsic incubation period table	Temperature-dependent	day	^52^
Mosquito infectivity	Decreasing with EIR	Person/bite	^55^
Human infectivity	Decreasing with human prevalence	Mosquito/bite	Assumed

**Table 5:** Parameters calibrated on the adult mosquito abundance.

Parameters	Values	Unit	Reference
Elasticity of larvae carrying capacity to rainfall	0.1 [0.1, 1]	dmnl	Calibrated
Elasticity of larvae failure to humidity	-0.34 [-2, -0.1]	dmnl	Calibrated
Elasticity of larvae survival to carrying capacity	0.25 [0.25, 2]	dmnl	Calibrated
Rainfall wash away to failure multiplier	1.39 [0.1, 2]	dmnl	^56^
Elasticity of oviposition to carrying capacity	1 [-1, 1]	dmnl	Calibrated
Elasticity of successful blood feeding to population density	-2 [-2, -0.1]	dmnl	Calibrated
Reference oviposition per day	5 [1, 6]	dmnl/day	^57^, calibrated
Reference successful blood feeding probability	1 [0.25-1]	dmnl	Calibrated
Maximum mosquito human ratio	2.91 [1, 10]	mosquito/person	Calibrated
Average time for mating (normal distribution)	3 [2, 4]	day	^50^
stdev of mating age	2 [0.5, 2]	dmnl	Calibrated
Gonotrophic cycle duration	5 [3, 5]	day	^44^, calibrated
Mosquitoes larvae to feeding carrying capacity ratio	1 [1, 10]	dmnl	Calibrated
Rainfall wash away threshold level	5 [5, 10]	mm/day	^56^
Rain accumulation time	3	day	Assumed

**Table 6:** Parameters calibrated on the *MB*-prevalence in adult mosquitoes.

Parameters	Values	Unit	Reference
Reference *MB* horizontal transmission rate (female-to-male)	0.6 [0.3, 1] (increased by *MB* level)	dmnl	^20^, Calibrated
Reference *MB* horizontal transmission rate (male-to-female)	0.8 [0.5, 1] (increased by *MB* level)	dmnl	^20^, Calibrated
Reference *MB* vertical transmission rate	0.5 [0.45, 1] (increased by *MB* level)	dmnl	^24^, Calibrated
Elasticity of *MB* vertical transmission rate to temperature	0.94 [0, 1]	dmnl	Calibrated
Elasticity of *MB* vertical transmission rate to NDVI	-0.5	dmnl	Calibrated
Elasticity of *MB* vertical transmission rate to humidity	0.25	dmnl	Calibrated
Elasticity of *MB* progress rate to temperature	4.48 [0, 5]	dmnl	Calibrated
Elasticity of *MB* progress rate to humidity	-0.22 [-1, 2]	dmnl	Calibrated

**Table 7:** Parameters for initialization.

Parameters	Values	Unit
Initial adult *MB* prevalence	0.072	dmnl
Initial adult population	566	mosquito
Initial indicated adult *MB* intensity distribution	[0-1] (decreasing with *MB*-intensity)	dmnl
Initial indicated pre-adult *MB* intensity distribution	[0-1] (decreasing with *MB*-intensity)	dmnl
Initial pre-adult *MB* prevalence	0.1	dmnl
Initial malaria prevalence	0.75	dmnl

**Table 8:** Parameter for releases and integrated strategies.

Parameters	Structure	Unit
Yearly mosquitoes release per capita	constant	mosquito/person
Release male fraction	constant	dmnl
Release age distribution table	Graphical function	dmnl
Mosquitoes release period duration	constant	day
Mosquito release stop time	constant	day
Release mating distribution		dmnl
Release *MB* distribution		dmnl
Release *Plasmodium* prevalence distribution		dmnl
Policy period start time	Constant	day
Intervention implementation coverage	Graphical function	dmnl
Intervention effectiveness duration		day

## References

[1] World Health Organization. *World malaria report 2025* (World Health Organization, 2025).

[2] Phillips, M. A. et al. Malaria. *Nat. Rev. Dis. Primers* **3**, 17050. 10.1038/nrdp.2017.50 (2017). 28770814 10.1038/nrdp.2017.50

[3] Lengeler, C. Insecticide-treated bed nets and curtains for preventing malaria. *Cochrane Database of Systematic Reviews* CD000363, 10.1002/14651858.CD000363.pub2 (2004). 10.1002/14651858.CD000363.pub2 15106149

[4] Tizifa, T. A. et al. Prevention efforts for malaria. *Curr. Trop. Med. Rep.* **5**, 41–50. 10.1007/s40475-018-0133-y (2018). 29629252 10.1007/s40475-018-0133-y PMC5879044

[5] Hemingway, J. et al. Averting a malaria disaster: Will insecticide resistance derail malaria control?. *Lancet* **387**, 1785–1788. 10.1016/S0140-6736(15)00417-1 (2016). 26880124 10.1016/S0140-6736(15)00417-1 PMC6215693

[6] Hoffmann, A. A. et al. Successful establishment of wolbachia in aedes populations to suppress dengue transmission. *Nature* **476**, 454–459. 10.1038/nature10356 (2011). 21866160 10.1038/nature10356

[7] Nazni, W. A. et al. Establishment of wolbachia strain walbb in malaysian populations of aedes aegypti for dengue control. *Current biology: CB* **29**, 4241-4248.e5. 10.1016/J.CUB.2019.11.007 (2019). 31761702 10.1016/j.cub.2019.11.007 PMC6926472

[8] Anders, K. L. et al. Reduced dengue incidence following deployments of *Wolbachia*-infected *Aedes aegypti* in Yogyakarta, Indonesia: A quasi-experimental trial using controlled interrupted time series analysis. *Gates Open Res.*10.12688/gatesopenres.13122.1 (2020). 32803130 10.12688/gatesopenres.13122.1 PMC7403856

[9] Frentiu, F. D. et al. Limited dengue virus replication in field-collected *Aedes aegypti* mosquitoes infected with *Wolbachia*. *PLoS Negl. Trop. Dis.*10.1371/journal.pntd.0002688 (2014). 24587459 10.1371/journal.pntd.0002688 PMC3930499

[10] Moreira, L. A. et al. A *wolbachia* symbiont in *aedes aegypti* limits infection with dengue, chikungunya, and *plasmodium*. *Cell* **139**, 1268–1278. 10.1016/j.cell.2009.11.042 (2009). 20064373 10.1016/j.cell.2009.11.042

[11] Walker, T. et al. The wmel *wolbachia* strain blocks dengue and invades caged *aedes aegypti* populations. *Nature* **476**, 450–455. 10.1038/nature10355 (2011). 21866159 10.1038/nature10355

[12] Bian, G., Xu, Y., Lu, P., Xie, Y. & Xi, Z. The endosymbiotic bacterium *wolbachia* induces resistance to dengue virus in *aedes aegypti*. *PLoS Pathog.* **6**, 1–10. 10.1371/journal.ppat.1000833 (2010). 10.1371/journal.ppat.1000833 PMC2848556 20368968

[13] Jeffries, C. L. et al. Novel *Wolbachia* strains in *Anopheles* malaria vectors from sub-Saharan Africa. *Wellcome Open Res.* **3**, 113. 10.12688/wellcomeopenres.14765.1 (2018). 30483601 10.12688/wellcomeopenres.14765.1 PMC6234743

[14] Baldini, F. et al. Evidence of natural *Wolbachia* infections in field populations of *Anopheles gambiae*. *Nat. Commun.*10.1038/ncomms4985 (2014). 24905191 10.1038/ncomms4985 PMC4059924

[15] Shaw, W. R. et al. *Wolbachia* infections in natural *Anopheles* populations affect egg laying and negatively correlate with *Plasmodium* development. *Nat. Commun.*10.1038/ncomms11772 (2016). 27243367 10.1038/ncomms11772 PMC4895022

[16] Gomes, F. M. et al. Effect of naturally occurring *Wolbachia* in *Anopheles gambiae* s.l. mosquitoes from Mali on *Plasmodium falciparum* malaria transmission. *Proc. Natl. Acad. Sci. U. S. A.* **114**, 12566–12571. 10.1073/pnas.1716181114 (2017). 29114059 10.1073/pnas.1716181114 PMC5703331

[17] Gomes, F. M. & Barillas-Mury, C. *Infection of anopheline mosquitoes with wolbachia: Implications for malaria control*10.1371/journal.ppat.1007333 (2018). 10.1371/journal.ppat.1007333 PMC6237385 30440032

[18] Chrostek, E. & Gerth, M. Is *Anopheles gambiae* a natural host of *Wolbachia*?. *mBio*10.1128/mBio.00784-19 (2019). 31186318 10.1128/mBio.00784-19 PMC6561020

[19] Mouillaud, T. et al. Limited association between *wolbachia* and *plasmodium falciparum* infections in natural populations of the major malaria mosquito *anopheles moucheti*. *Evol. Appl.* **16**, 1999–2006. 10.1111/eva.13619 (2023). 38143905 10.1111/eva.13619 PMC10739076

[20] Nattoh, G. et al. Horizontal transmission of the symbiont microsporidia mb in *Anopheles arabiensis*. *Front. Microbiol.*10.3389/fmicb.2021.647183 (2021). 34394019 10.3389/fmicb.2021.647183 PMC8355901

[21] Akorli, J. et al. Microsporidia mb is found predominantly associated with anopheles gambiae s.s and anopheles coluzzii in ghana. *Scientific Reports* **11**, 10.1038/s41598-021-98268-2 (2021). 10.1038/s41598-021-98268-2 PMC8452686 34545153

[22] Nattoh, G. et al. Microsporidia mb in the primary malaria vector *anopheles gambiae* sensu stricto is avirulent and undergoes maternal and horizontal transmission. *Parasit. Vectors* **16**, 335. 10.1186/s13071-023-05933-8 (2023). 37749577 10.1186/s13071-023-05933-8 PMC10519057

[23] Bukhari, T., Pevsner, R. & Herren, J. K. Microsporidia: A promising vector control tool for residual malaria transmission. *Front. Trop. Dis.*10.3389/fitd.2022.957109 (2022).

[24] Herren, J. K. et al. A microsporidian impairs *Plasmodium falciparum* transmission in *Anopheles arabiensis* mosquitoes. *Nat. Commun.*10.1038/s41467-020-16121-y (2020). 32366903 10.1038/s41467-020-16121-y PMC7198529

[25] Mfangnia, C. N. T., Tonnang, H. E. Z., Tsanou, B. & Herren, J. Mathematical modelling of the interactive dynamics of wild and microsporidia mb-infected mosquitoes. *Math. Biosci. Eng.* **20**, 15167–15200. 10.3934/mbe.2023679 (2023). 37679176 10.3934/mbe.2023679

[26] Boanyah, G. Y., Koekemoer, L. L., Herren, J. K. & Bukhari, T. Monitoring the capacity of microsporidia mb transgenerational spread in *Anopheles arabiensis* populations. *Insects*10.3390/insects16121206 (2025). 41465646 10.3390/insects16121206 PMC12733993

[27] Boanyah, G. Y., Koekemoer, L. L., Herren, J. K. & Bukhari, T. Effect of microsporidia mb infection on the development and fitness of *anopheles arabiensis* under different diet regimes. *Parasit. Vectors* **17**, 294. 10.1186/s13071-024-06365-8 (2024). 38982472 10.1186/s13071-024-06365-8 PMC11234536

[28] Ahouandjinou, M. J. et al. First report of natural infection of *Anopheles gambiae* s.s. and *Anopheles coluzzii* by Wolbachia and microsporidia in Benin: A cross-sectional study. *Malar. J.* **23**, 72. 10.1186/s12936-024-04906-1 (2024). 38468292 10.1186/s12936-024-04906-1 PMC10926679

[29] Moustapha, L. M. et al. Spatial distribution of microsporidia mb along clinal gradient and the impact of its infection on pyrethroid resistance in *Anopheles gambiae* s.l. mosquitoes from Nigeria and Niger Republic. *Parasitologia*10.3390/parasitologia5030031 (2025).

[30] Moustapha, L. M. et al. First identification of microsporidia mb in *Anopheles coluzzii* from Zinder city, Niger. *Parasit. Vectors* **17**, 39. 10.1186/s13071-023-06059-7 (2024). 38287334 10.1186/s13071-023-06059-7 PMC10826271

[31] Homer, J. B. & Hirsch, G. B. System dynamics modeling for public health: Background and opportunities. *Am. J. Public Health* **96**, 452–458 (2006). 16449591 10.2105/AJPH.2005.062059 PMC1470525

[32] Enos, J. R. & Schott, R. *& Schott, E* (A system dynamics approach, 2015).

[33] Ritchie-Dunham, J. L. & Galván, J. F. M. Evaluating epidemic intervention policies with systems thinking: A case study of dengue fever in Mexico. *Syst. Dyn. Rev.* **15**(2), 119–138 (1999).

[34] Subyan, A. N., Jabar, N. F. A. A., Darlynie, C. R. & Ahmad, N. Dengue outbreak: A system dynamics approach. *Journal of Technology Management and Business* **5**, 10.30880/jtmb.2018.05.01.007 (2018).

[35] Jaafar, I. A., Abidin, N. Z. & Jamil, J. M. Modelling the prediction of dengue outbreak using system dynamics approach. *J. Teknol.* **78**, 107–113. 10.11113/jt.v78.8984 (2016).

[36] Blanco, S. M. Statics and dynamics of malaria transmission: The relationship between prevalence in humans and mosquitoes. *Fortune Journal of Health Sciences* **5**, 374–406 (2022).

[37] Pedercini, M., Movilla Blanco, S. & Kopainsky, B. Application of the malaria management model to the analysis of costs and benefits of DDT versus non-DDT malaria control. *PLoS ONE* **6**, e27771. 10.1371/journal.pone.0027771 (2011). 22140467 10.1371/journal.pone.0027771 PMC3227603

[38] Mfangnia, C. N. T., Tonnang, H. E. Z., Tsanou, B. & Herren, J. K. An eco-epidemiological model for malaria with microsporidia mb as bio-control agent. *Model. Earth Syst. Environ.* **11**, 221. 10.1007/s40808-025-02322-1 (2025). 40255466 10.1007/s40808-025-02322-1 PMC12003625

[39] Affognon, S. B. et al. Optimizing microbe-infected mosquito release: A stochastic model for malaria prevention. *Front. Appl. Math. Stat.*10.3389/fams.2024.1465153 (2024).

[40] Hoffmann, A. A. et al. Successful establishment of wolbachia in aedes populations to suppress dengue transmission. *Nature* **476**, 454–457. 10.1038/nature10356 (2011). 21866160 10.1038/nature10356

[41] Walker, T. et al. The wmel wolbachia strain blocks dengue and invades caged *aedes aegypti* populations. *Nature* **476**, 450–453. 10.1038/nature10355 (2011). 21866159 10.1038/nature10355

[42] Vásquez, V. N., Kueppers, L. M., Ravsić, G. & Marshall, J. M. wmel replacement of dengue-competent mosquitoes is robust to near-term change. *Nat. Clim. Chang.* **13**, 848–855 (2023). 37546688 10.1038/s41558-023-01746-w PMC10403361

[43] Beier, J. C. et al. Integrated vector management for malaria control. *Malar. J.*10.1186/1475-2875-7-S1-S4 (2008). 19091038 10.1186/1475-2875-7-S1-S4 PMC2604879

[44] Maharaj, R. Life table characteristics of *anopheles arabiensis* (diptera: Culicidae) under simulated seasonal conditions. *J. Med. Entomol.* **40**, 737–742. 10.1603/0022-2585-40.6.737 (2003). 14765646 10.1603/0022-2585-40.6.737

[45] Klowden, M. J. Blood, sex, and the mosquito (1995).

[46] Dahalan, F. A., Churcher, T. S., Windbichler, N. & Lawniczak, M. K. The male mosquito contribution towards malaria transmission: Mating influences the anopheles female midgut transcriptome and increases female susceptibility to human malaria parasites. *PLoS Pathog.*10.1371/journal.ppat.1008063 (2019). 31697788 10.1371/journal.ppat.1008063 PMC6837289

[47] Maina, T. et al. Maximizing horizontal transmission through mating: Increased mating frequency and mating competitiveness associated with microsporidia mb-infected *anopheles arabiensis* males. *Malar. J.* **24**, 114. 10.1186/s12936-025-05354-1 (2025). 40205501 10.1186/s12936-025-05354-1 PMC11983955

[48] Nicoletti, M. *Three scenarios in insect-borne diseases*, 99–251 (Elsevier, 2020).

[49] Ashley, E. A. & White, N. J. *The duration of plasmodium falciparum infections*10.1186/1475-2875-13-500 (2014). 10.1186/1475-2875-13-500 PMC4301960 25515943

[50] Tripet, F., Thiemann, T. & Lanzaro, G. C. Effect of seminal fluids in mating between M and S forms of anopheles gambiae. *J. Med. Entomol.* **42**, 596–603 (2005). 16119548 10.1093/jmedent/42.4.596

[51] Oliva, C. F., Benedict, M. Q., Lempérière, G. & Gilles, J. Laboratory selection for an accelerated mosquito sexual development rate. *Malar. J.* **10**, 135 (2011). 21595988 10.1186/1475-2875-10-135 PMC3120732

[52] Beier, J. C. Malaria parasite development in mosquitoes. *Annu. Rev. Entomol.* **43**, 519–543 (1998). 9444756 10.1146/annurev.ento.43.1.519

[53] Depinay, J. M. O. et al. A simulation model of african anopheles ecology and population dynamics for the analysis of malaria transmission. *Malaria Journal* **3**, 10.1186/1475-2875-3-29 (2004). 10.1186/1475-2875-3-29 PMC514565 15285781

[54] Lyons, C. L., Coetzee, M. & Chown, S. L. Stable and fluctuating temperature effects on the development rate and survival of two malaria vectors, anopheles arabiensis and anopheles funestus. *Parasites & Vectors* **6**, 104. 10.1186/1756-3305-6-104 (2013). 23590860 10.1186/1756-3305-6-104 PMC3637585

[55] Smith, D. L., Drakeley, C. J., Chiyaka, C. & Hay, S. I. A quantitative analysis of transmission efficiency versus intensity for malaria. *Nat. Commun.*10.1038/ncomms1107 (2010). 21045826 10.1038/ncomms1107 PMC3065713

[56] Paaijmans, K. P., Wandago, M. O., Githeko, A. K. & Takken, W. Unexpected high losses of anopheles gambiae larvae due to rainfall. *PLoS One*10.1371/journal.pone.0001146 (2007). 17987125 10.1371/journal.pone.0001146 PMC2063461

[57] Afrane, Y. A., Zhou, G., Lawson, B. W., Githeko, A. K. & Yan, G. Life-table analysis of anopheles arabiensis in western kenya highlands: Effects of land covers on larval and adult survivorship. *American Journal of Tropical Medicine and Hygiene* **77**, 660–666. 10.4269/ajtmh.2007.77.660 (2007). 17978067

